# Our Correspondence

**Published:** 1876-07

**Authors:** 


### Our Correspondence.

### FLORIDA LETTER.

SOUTH SIDE LAKE JESSUP, FLA.,

March 25th, 1876.

*Dear Doctor* :—Six years ago a party of four of us from the north spent a winter in this locality. We are about two hundred and thirty miles south of Jacksonville. We found the climate the most desirable here, and our present experience only confirms our former opinion.

We are two miles from the shore of the lake, and eighty feet above its level. So we are on very high ground for this State. A short distance to the south of us the water runs east in part, and south to lake Ocheechobe. So we are on the divide, on the high land of all this region of country. Dr. Henry Foster, of Clifton Springs Sanitarium, is our host. He has built a nice large house here, on the shore of a beautiful little lake about the size of lake Eldridge in the Park at Elmira. His yard has some three acres of enclosed native forest.

His grounds contain the Live Oak, Water Oak, Palmetto, Hickory, Ash, Magnolia, Sweet and Sour Orange. He will soon set out the Pecan, the English Walnut, and other semi-tropical trees and shrubs, besides many flowering trees and vines. He will grade a road-way around lake Charm and set out the Australian Eucalyptus to beautify the drive-way, and secure at the same time the benefit of its anti-malarious properties He has set out some twenty acres to orange trees, and they are growing finely. He will in a few years have one of the finest groves in the State. He has put up an engine some six hundred feet from the lake, and a large force pump, and distributed about three miles of iron pipe through his grove and about the grounds. He can water his entire premises, reaching every tree, in about six hours, throwing in that time some twelve hundred barrels of water. Trees often suffer here for want of rain. The doctor can now supply the element needed to keep his trees growing all the time.

This section has improved very much since my former visit. Where there were but ten orange trees set out, there are now thousands. This part of the State is soon to furnish a large quantity of fruit for our Northern market of the very finest quality. It was formerly thought that the orange tree would only do well in the low rich hammock grounds. But experience has proved that the higher lands are the best. For when the tap root of the tree reaches the water, it rots and the top of the tree begins to die out. Land in the wild state that is fit for orange growing, now sells from fifty to one hundred dollars per acre.

This section has had but little rain for the past year. The streams and lakes are from two to six feet lower than usual. Navigation in the upper St. John's river has been seriously impeded. Boats that draw more than about two feet of water cannot get up the river higher than Enterprise. Boating beyond lake Harney has been pretty much abandoned. Never saw so much dry land here before.

We are now eating new potatoes, string beans, peas fresh from the vines, lettuce, turnips, beets and radishes of the finest quality. They can raise from two to three crops in a year on the same ground. We have very fine beef, the finest of venison and wild turkey, which is really delicious. We get also plenty of quail to add to the variety of our cuisine.

The weather has been so warm that we have not hunted for the larger game, only for quail and small birds. The marshes are so dry that all the ducks have left them, and also most of the water birds that usually haunt this region. The large game has been hunted out pretty close in this vicinity. Yet the other day a fine deer came within gun shot of our door. We were not there at the time, so could not send him our compliments, which we regretted very much. This country is infested with fleas, wood-ticks, sand-flies, cockroaches, huge house flies, nearly as large as a humble-bee, and musquitoes. One has to scratch to live, and live to scratch. But it keeps one lively and industrious, no time runs to waste.

Malarious forms of disease prevail in this section and indeed all over the State. They are of a mild type and not dangerous. They are amenable to quinine in small doses. There is also some rheumatism here; think it comes more from want of proper clothing and the common necessaries of life. The inhabitants are as yet very poor, live mostly in log huts, no windows and scanty clothing, and are limited to very few articles of diet. They use mostly hot bread, made either of wheat flour or corn meal, bacon, rice, hominy and villianous coffee without milk or sugar. They are usually tall, lank, lean, pale, cadaverous looking specimens of humanity. But they live in hopes of being better off when their orange trees come into bearing, which many of them will soon do. This State, in fact, is worth but little else than for orange growing—climate as a winter resort—fish and game, besides its lumber which it furnishes in large quantities. It is pretty much, as the girl said, the inhabitants "live on sweet potatoes in the summer and strangers in the winter."

Saw the other day at the wharf on lake Jessop, a huge alligator in a cage made of poles and bark. He was twelve feet in length and would weigh from five to six hundred pounds. He was captured some fifty miles south of here and transported over land on a wagon. He was caught by a noose made of rope. He was hitched to a tree to remain over night. He broke a rope doubled that was three-fourths of an inch in diameter—but was re-captured the next day. He was bound for the Centennial—himself doubtless a centenarian. The man who secured him has orders to procure two more and forward them to Philadelphia. If one wants to get a sight of alligators, let him take a dog in a boat, and make him bark, and they will come about from all quarters, hoping to make a meal of the dog, of which they devour many every year. Many a hunter has said something beside the first sentence of the doxology when his favorite dog has been captured by one of these dog-traps. They often exhibit some cunning. One hunter told me that they will lie in wait at some deer crossing on the streams—let the deer pass and catch the dogs. He told me one caught five dogs in a single day in that manner. Is not that strategy? Is not that hard on dogs? Some years since a firm in New England paid one dollar for the skins. They were slaughtered by the hundred—but they did not make as good leather as was anticipated, so the alligators have peace again. Only wish they could devour about one thousand cur dogs that infest our city.

Since my visit here five years ago, there has been much done to improve the beauty of the scenery up the river. Very many neat little cottages have been erected and a large number of orange groves started. Men from the north have purchased some groves and made winter homes. Met one man from Chicago who has purchased seven thousand acres and is setting out a very large grove. One man went into Orange Co. seven years since, with a capital of one hundred and forty dollars. He was offered this year *thirty thousand dollars* for his grove. Pretty good business for the capital invested and seven year's labor.

Groves are selling from five thousand to fifty thousand dollars. Early vegetables and oranges are the main resources at the present of this state.

The climate is truly charming. We enjoy the clear air—the almost perpetual sunshine—while you are amid sleet, snow, wind and cold.

Yours very truly,

S. O. GLEASON.

UNION CITY, May 11th, 1876.

DR. UP DE GRAFF:

Enclosed find subscription for BISTOURY. I wish to ask you to publish in your next issue a prescription for the cure of an affection, an intolerable annoyance, to-wit: an itching of the anus, especially when I lie down. R. S. F.

The difficulty complained of is one of exceeding prevalence, and often provocative of the keenest discomfort. There exists severel forms of the complaint. One, produced by a little parasite, called a "pin worm," which deposits its eggs about once in four or six weeks, in the folds of the sphincter muscles, outside the body. At such time the itching or tickling sensation is almost intolerable. This difficulty is readily curable by annointing the parts, regularly, every night, with sweet oil, injecting a small quantity with a syringe, within the anus. This application destroys the worm, as it cannot breathe, or use its spiracles, when immersed in oil. By continuing the treatment for several months, each succeeding brood, as they are hatched, are destroyed by the oil, and the patient is at last relieved from his troublesome visitors.

Another form of this affection is the one known as *pruritus*, accompanied with a peculiar form of itching, usually aggravated by walking or when the body is kept warm, in bed. It resembles *eczema*, in that the anus is covered with very minute vesicles, which rupture and discharge an irritating fluid that covers the parts and produces little fissures or cracks which are exceedingly difficult to heal, and are extremely annoying, by reason of the constant itching, and sometimes burning or smarting in the parts. The cause seems to be very obscure. Usually is present as an accompaniment of dyspepsia, constipation or other irregularities of the digestive canal. A salve, made of cosmoline, one ounce, and acetate of morphia ten to fifteen grains, thoroughly mixed, and smeared over the parts affected every night, will afford great relief.

This application will often heal the affection, but the patient will suffer a relapse now and then in spite of all medication.

---

THE GREAT ENGLISH PHYSICIAN AGAIN.—Our readers will remember our criticisms upon a traveling mountebank who flourished in these parts about the month of April. So thoroughly convinced were we that he was an ignorant pretender, that we took some trouble to enquire into his history, with the result given below. In view of the wonderful pretentions and ostentatious display of this impudent quack, and the considerable patronage he secured from citizens who are accredited with common sense, the showing is a bad one. We were informed that the "Great English Physician" had been an auctioneer in Newark, Ohio, that he sold out his portion of the business to his brother and invested the proceeds in his "anatomical cabinet," and without any other preparation than that gleaned from Dr. Gleason's book, commenced his grand lecturing tour, as the "Great English Physician, from London, England." These facts are substantiated by the following letters from three respectable citizens of Newark, Ohio, two being merchants of that city and another a banker:

NEWARK, OHIO, April 15th, 1876.

DR. T. S. UP DE GRAFF:

*Dear Sir:*—In reply to yours of the 12th inst. Several years ago two brothers, Jacob V. Burner and Henry R. Burner, kept an auction store in this city, and for a short time went to Columbus Ohio, where they seem to have dissolved partnership, and separated. J. V. Burner returned here, re-opened his auction store, and carried it on until a few days ago, and is here now in another business.

The younger brother HENRY R. BURNER is now, and has been for several years, a traveling lecturing physician, and is said to be, at present, in New York State, or thereabouts.

C. H. N.

NEWARK, OHIO, APRIL 15th, 1876.

DR. UP DE GRAFF:

In reply we would say, that J. V. Burner and H. R. BURNER have been, for a number of years, until lately, engaged in the auction business in this city. Mr. H. R. Burner we think at this time is in your immediate vicinity, somewhere near Elmira, and is now a "traveling doctor."

E. F.

NEWARK, OHIO, APRIL 16th, 1876.

Dr. UP DE GRAFF:

Mr. H. R. Burner was formerly in the auction business in this city, and also in Columbus, O. I have known him for twenty years, and never knew or heard of his having been to England, or anywhere else, to seek a medical education. Think the only education he has of *any* sort, is what he obtained in the auction room. We take no stock in him here as a "doctor." B.

And so we "rest our case," making only the single observation, that traveling doctors, as a class, are "frauds." We have known of but two exceptions to this rule, and "The Great English Physician" is not one of them. We give below an item taken from the Elmira Daily *Gazette*, and written by Rev. T. K. Beecher in his Saturday Miscellany, which covers the ground quite effectually:—

"People should not forget that their physician or surgeon is fighting a losing battle. It is appointed unto all men once to die—doctor or no doctor. Sooner or later all doctors and all surgeons are to be worsted. They fight against an enemy that can never be defeated. For this reason all our medical advisers are entitled to considerate treatment—more considerate than they usually receive.

"The most that can be asked of surgeons and physicians, is that they stand by their work, and slowly but surely win the confidence of their fellow men by successes as numerous as can be reasonably expected. When one patient recovers it does not by itself prove that the doctor has been skillful. When a patient dies, the fact by itself does not prove the doctor unskillful. Patients vary quite as much as doctors. Only by a term of years can it be shown that one doctor is wise and another unwise. That surgeon or physician alone is worthy of confidence, therefore, who plants himself in one place, and consents to be judged largely.

"—As a rule, all traveling doctors are to be discountenanced. The presumption is that they are adventurers and quacks. They come into our city, advertise largely, and it is nobody's business to examine their credentials. They promise profusely; take pay in advance; guarantee nothing; stay by just long enough to play their game, and then move on.

"It is conceivable that a man may have gained remarkable skill in the treatment of certain diseases; and, wishing to do the utmost good, or earn most money, may travel. But in this fraudful world such cases are rare. The rule holds: All traveling doctors are to be accounted quacks and swindlers, until by indisputable credentials, or by transparent modesty and truthfulness, they prove themselves trustworthy."

---

HARRISBURG, PA., May 10, 1876.

DR. UP DE GRAFF:

We have a little child—a girl—aged twelve years, whose spine begins to turn to the left, forming quite a bend. We think she grows worse, and we find too that her strength and health is failing. Can anything be done to stop the disease?

J. P.

Yes. Take her to a surgeon and have a suitable brace fitted to her. Or, better still, place her in an institution where such ailments are specially treated. See BISTOURY of October, 1873, for a fully illustrated article upon spinal curvature, and means of cure.

---

CEDAR KEYS, Fla.,
April 15, 1876.

DR. T. S. UP DE GRAFF:

*Dear Sir:*—I send for your inspection and opinion regarding its character, with the causes that may have combined to produce it, a substance which was removed from the Sublingual Gland of a man named D. O. For some days previous to its removal, he had complained of feeling some hard substance underneath the tongue, but attaching no importance to it, almost forgot its existence. Finally its presence becoming a source of irritation, he applied to me for aid. On inspection, I found a slightly pointed tumor underneath the tongue, the apex presenting a whitish appearance. The touch of a lancet and light pressure of the fingers disgorged the queer specimen I send you. No irritation of tissues immediate to, or surrounding the tumor, existed, neither did suppuration or discharge of any kind follow its removal. You will perceive that I have been testing its density and hardness with my knife. The reverse side of the oval presents its features when removed. Mr. O. is a farmer by occupation, is temperate in every respect, enjoys excellent health, and is a strong, athletic man. His age is about forty-five years, has never had disease of the kidneys or bladder, nor to his knowledge passed gravelous deposits. Without hazarding an opinion respecting its character, I forward it to you for inspection.

Very Respectfully, &c.,
G. A. P., M. D.

The specimen sent is a yellowish, hard concretion, about the size of two peas, placed side by side, and is a calculus, formed from the secretions of the sub-maxilliary gland. It is termed *ranula*. The specimen is a very handsome one, which we have placed in our cabinet, and for which we return thanks.

---

AMENIA, N. Y., May 10th, 1876.

EDITOR BISTOURY:

*Dear Sir:*—A druggist of this place handed me this morning a copy of the Jan. No. of your Quarterly Journal, and called my attention to the case of Geo. M. Ball, with a "Hooked Adder" in his stomach, or from whose stomach a "Hooked Adder" was reported to have been ejected. I am personally acquainted with said Ball and all the facts in the case. Ball's report, and exhibition of a large Hooded Adder, two feet eight inches in length was indeed a hoax. But I do not consider a "Country or City Editor" as demented because he copies from another journal, for instance, the Springfield Republican, any more than I do the "Quarterly Journal or BISTOURY." But I have far greater respect for either of them than I have for a journal which attempts by *bravado* and *bullying* to suppress and crush out facts of the most vital importance to mankind in general.

Such as the possibility of a snake, toad, lizard and many others of the reptile race, getting within and living in the human stomach, and maintaining life therein. Your representation that the creatures have pulmonary organs, (lungs), and therefore must have airification is *correct*. But when you announce that they cannot live in any part of the alimentary *canal*, your stolidity is only equaled by your pomposity. The most ordinary student in physiology will inform you that the atmosphere or air acts upon every portion of the mucous membrane throughout the entire body. Thus air is supplied. Again, the gastric juice is not inimical (preservative) of life. And so of all the other natural gasses and juices of the stomach, else the stomach itself would be destroyed, and life would be a failure. Again, your assertion that worms, or entozœ, adapted to living in closed cavities, cannot live in the stomach, but are always found in the bowels, is incorrect. The Taenia Solum, (tape worm), as well as the Ascaris Lumbricoides, (long round worm), are said by Prof. Dalton to be the result, in Taenia Solum, of tuberculous developments in meats hatched in the stomach. And in Lumbricoides, of eggs drank in water and hatched in the stomach. Again it is allowed that they may eminate through the whole length of the alimentary canal. It is equally certain that they may remain in the stomach for months and years at a time.

I have removed hundreds of tape worms within the last ten years, and from the general tenor, or complaint of symptoms with patients, have no doubt that in every instance the worm was in the stomach, though a portion of the worm may have been lying in the bowels, as no doubt they do sometimes.

Claravoyance demonstrates, or reveals the fact that the worms occupy the stomach, as well as the other fact that accidental paracites occupy it also. It makes no difference whether you recognize claravoyance or no. One demonstrated fact outweighs all your assertions and dogmatisms, because facts are God's eternal truths; or the results of general principles revealed through infinite unfoldments in the realms of matter. Science is the revealment or revalation of facts susceptible of being taught, or revealed from one to another in a manner useful and intelligent.

Now give a fact, (a patient inhabited of an eel), but denying the possibility, how long would it take you scientifically to cure the patient? Eternity would not help you to take the first progressive steps towards a cure. Admit the possibility, address yourself with means adequate to end, the cause will be removed and patient cured, nobody harmed but great good done.

In conclusion, I will give you a few facts for contemplation. In the last eight years and a half, I have removed from out the human system, five snakes, two eels, two toads, one bull-head and nineteen lizards, and still they come.

One of the eels is fifteen inches long, one inch in diameter, and weighs three ounces; was extracted from the bowels of my patient in the presence of two witnesses besides the person extracting and the patient—three unimpeachable witnesses beside the patient herself. One snake nine and one-quarter inches in length, and alive, was vomited off my patients stomach in the presence of two witnesses, where no diagnosis of snake or any other paracite creature had been given, so no one was looking for snake. One tree toad vomited out of a child's stomach, (child 4 years, 9 months and 20 days old), alive, while in her mother's arms; no diagnosis of toad or paracite had been given.

Now in view of all these facts, what will you say about it? Make a duced fool of yourself; or about ship in the woods of your ignorance of the subject and begin anew and make something out of yourself; or *spoil* for the want of common honesty and candor? The decision and its consequences are yours, and make the most you can of them.

Very Res·ectfully Yours,

H. C. C., M. D., Otis, Mass.

P. S.—I once practiced in Owego from '51 to '63. I am known in Binghamton and Elmira, and all along the E. R. R., as the Worm Doctor. I hardly suppose you will publish my communication, but do as you like about it.

Respectfully, H. C. C.

Hail Columbia! What *can* we say to such an effusion as the foregoing? What needs to be said to a man who can remove snakes, eels, toads, lizards and "bull heads" from the "human system." What is the use of saying anything, where all this is done in the presence of "three unimpeachable witnesses" Then, when the length of the child and the age of the tree toad is given, or *vice versa*, the statement is so convincing that no room is left for argument.

The P. S. too, is so overwhelming—showing as it does, the evident reputable standing of this eminent "worm doctor," that any effort at contradiction must of necessity prove futile. Yet, we must hazard this modest opinion, that no frog, lizard, snake, or other *Batrachian*, can exist, alive, for a single day, even, anywhere in the alimentary canal of man or beast. The respiration of these creatures is not confined to the lungs alone, for the blood seeks the capillaries of the skin for aeration by the atmosphere, and without this assistance to the lungs, the animal cannot live for any considerable time. Take this "worm doctor's" toad, that was expelled from that child of 4 years, 9 months, 20 days—(the minutes and seconds, we regret, are not given, upon which much of the accuracy of the statement must hinge), and smear it with oil, when it will die almost as speedily as though deprived of its lungs. What would happen to it were it confined within the stomach and coated with the unctuous secretions of that organ? If the stomach and bowels were dry and inflated, or as empty as the "worm doctor's" cranium, perhaps his menagerie of reptiles might flourish therein, to add strength to his statements, and, perhaps, further reputation to his skill as a "reptile doctor."

Briefly, reptiles *cannot* live in the stomachs of men. They must breathe, and the stomach does not contain sufficient air to sustain life, and if it did, even then, the reptile must die from the inunction it would receive from the secretions of the stomach. Again, the elevation of temperature, in the surroundings of all cold-blooded animals, augments the demand for air, increases the activity of their respiration, in which they exhale large quantities of carbonic acid gas, in which they must necessarily perish, in so close a receiver as would be the stomach. When the "worm doctor" secures a patient with a wood-chuck in his stomach, we know of two riflemen on east hill, in this city, that will sit up with that patient a reasonable time, for the felicity of knocking off his head, at a hundred yards—the wood-chuck is meant, of course, but you may include the doctor rather than have any further argument about the matter.

---

☞ Several correspondents are notified that we do not furnish specimen copies of those journals with which we club, neither will we send, in future, specimen copies of the BISTOURY, unless a two cent stamp is sent, to prepay the postage of every copy ordered.

